# The influence of lightning induced voltage on the distribution power line polymer insulators

**DOI:** 10.1371/journal.pone.0172118

**Published:** 2017-02-24

**Authors:** Mahdi Izadi, Muhammad Syahmi Abd Rahman, Mohd Zainal Abidin Ab-Kadir, Chandima Gomes, Jasronita Jasni, Maryam Hajikhani

**Affiliations:** 1 Centre for Electromagnetic and Lightning Protection Research (CELP), Faculty of Engineering, Universiti Putra Malaysia, Selangor, Malaysia; 2 Electrical Engineering Department, Islamic Azad University Firoozkooh Branch, Firoozkooh, Iran; Texas A&M Univ, UNITED STATES

## Abstract

Protection of medium voltage (MV) overhead lines against the indirect effects of lightning is an important issue in Malaysia and other tropical countries. Protection of these lines against the indirect effects of lightning is a major concern and can be improved by several ways. The choice of insulator to be used for instance, between the glass, ceramic or polymer, can help to improve the line performance from the perspective of increasing the breakdown strength. In this paper, the electrical performance of a 10 kV polymer insulator under different conditions for impulse, weather and insulator angle with respect to a cross-arm were studied (both experimental and modelling) and the results were discussed accordingly. Results show that the weather and insulator angle (with respect to the cross-arm) are surprisingly influenced the values of breakdown voltage and leakage current for both negative and positive impulses. Therefore, in order to select a proper protection system for MV lines against lightning induced voltage, consideration of the local information concerning the weather and also the insulator angles with respect to the cross-arm are very useful for line stability and performance.

## Introduction

Lightning affects the performance of power lines by both direct and indirect effects where the transient high voltages may cause flashover on the electrical equipment on the power line. Direct lightning strikes, may intercept with line conductors, towers or shielding wires. The probability of direct strike in a given region increases with line height, thus, high voltage (HV) lines may subject to direct strikes more than medium voltage (MV) or low voltage (LV) lines.

On the other hand, when lightning strikes the ground or any object close to a line, the electromagnetic fields will propagate in all directions. The inductive and capacitive coupling of such electromagnetic fields with conducting wires induce voltage impulses in the power system [1–5]. These lightning induced overvoltage (LIOV) may cause significant problems in MV and LV power lines because of the low value of critical flashover (CFO) voltage compared to that of HV line [5]. Moreover, the chance of an indirect effect is higher than that of a direct strike as for any lightning event around a power line, LIOV can appear on the power line.

The high lightning ground flash density makes Malaysia more vulnerable to both direct and indirect lightning effects on power systems. In fact, many parts of Malaysia experience up to 200 thunderstorm days per year which serves as one of the countries with a very high flash density in the world. Local electrical utility, TNB, has claimed that about 50% of total failures in their system are caused by lightning strikes [6]. Therefore, it is necessary to consider the effect of lightning on most electrical components, including insulators.

The selection of powerline insulators should be done after thorough investigation on the weather conditions of a particular region and voltage conditions of the system [7–12]. The configuration of the line and the radial distance between conductors with respect to the ground may also affect the electric field distribution along an insulator and the value of the breakdown voltage under various conditions [11–13].

The combined influence of the weather conditions and the inclination of insulators with respect to the cross arm have not been studied in detail in the literature. Unintended and undesired inclination of isolators in real MV power lines have been reported from many parts of Malaysia (personal communication with TNB Officers). Such distortions may be due to the forces act on an insulator as a result of cornering or external forces such as fallen trees, high wind shear with excessive rainfall etc. Therefore, the electrical performance of an insulator will change and this should be considered in the design or modification of line. To address this issue, in this paper, the electrical behaviour of a 10 kV polymer insulator under an impulse voltage (due to lightning induced voltage) was considered and the effects of weather conditions and inclination of insulator on the power line performance under LIOV has been investigated. Polymer insulator has been considered for the analysis due to the wide-usage of such insulators based on their many advantages in power system applications.

## Material and methods

### Lightning induced voltage

Several works have shown interest in lightning induced voltage over the past few decades. For example, a group of Japanese researchers [14] studied induced voltage waveforms while simultaneously measuring lightning stroke current waveforms for both positive and negative polarities. Three continuous years of study has demonstrated the behaviour of induced voltages along a length of distribution line. A reduced scale work was conducted by [15] to simulate a lightning channel with consideration of a lossy ground in order to evaluate lightning induced voltage on overhead lines. Meanwhile, a study by [16] focused on the effect of voltage attenuation on shielded wires and surge arrestors due to lightning induced voltage. To date, works for the evaluation of lightning induced voltage by numerical methods have become more popular. Regarding the simple Rusck formula, some elaborate models e.g. [17–19] have been developed. In addition, as presented by Yutthagowith et al., the Cooray-Rubinstein expression has been used for the calculation of lightning induced voltage due to a flat ground and a tall structure flash [20].

**Fig 1:**
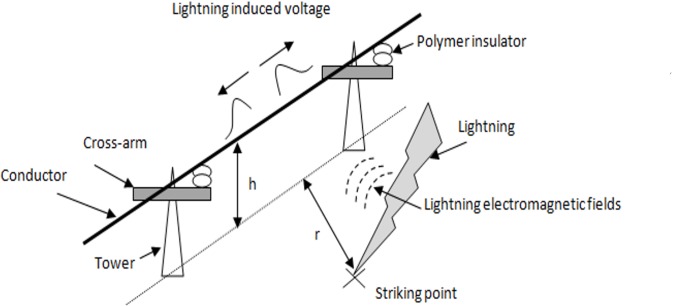
Geometry of problem. Lightning strike at a distance eventually coupled with the power line causing lightning induced voltage to occur in the system.

A lightning induced voltage can be created on the power line by coupling between the lightning electromagnetic fields and the power line [1, 3, 4], as shown in Fig 1.

In order to evaluate the lightning induced voltage on the power line, the lightning current wave shape of the channel base (striking point and at different heights along the channel) should be modelled. In this study, the channel base current was simulated using the sum of two Heidler functions as expressed by Eq (1) and the typical current parameters are listed in Table 1 as follows [1, 3];

$$\text{i}\left(0,\text{t}\right)=\left[\frac{{\text{i}}_{01}}{\eta_{1}}\frac{{\left(\frac{\text{t}}{{Г}_{11}}\right)}^{{\text{n}}_{\text{c}1}}}{1+{\left(\frac{\text{t}}{{Г}_{11}}\right)}^{{\text{n}}_{\text{c}1}}}\exp\left(\frac{-\text{t}}{{Г}_{12}}\right)+\frac{{\text{i}}_{02}}{\eta_{2}}\frac{{\left(\frac{\text{t}}{{Г}_{21}}\right)}^{{\text{n}}_{\text{c}2}}}{1+{\left(\frac{\text{t}}{{Г}_{21}}\right)}^{{\text{n}}_{\text{c}2}}}\exp\left(\frac{-\text{t}}{{Г}_{22}}\right)\right]$$ (1)

Where

i_01_,i_02_ are the amplitudes of the channel base current,

Γ_11_,Γ_12_ are the front time constants,

Γ_21_,Γ_22_ are the decay- time constants,

n_c1_,n_c2_ are the exponents (2~10),

η1=exp⁡[−(Γ11Γ12)(nc1Γ12Γ11)1nc1],

η2=exp⁡[−(Γ21Γ22)(nc2Γ22Γ21)1nc2].

**Table 1:** The channel base current parameters based on the sum of two Heidler functions [3].

i_01_ (kA)	i_02_ (kA)	Γ_11_ (μs)	Γ_12_ (μs)	Γ_21_ (μs)	Γ_22_ (μs)	n_1_	n_2_	λ (m)
19.5	12.3	1	2	8	30	2	2	1500

**Fig 2:**
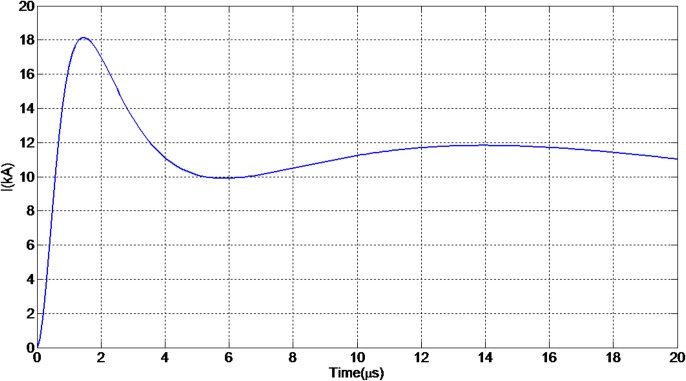
The channel base current waveshape. Modelled using the MTLE current model to study the effect at different heights.

Fig 2 shows the channel base current with a current peak of 18.1 kA and a time to peak of about 1.5 μs [3].

In this study, the current waveshape at different heights along the channel was modelled using the MTLE current model as expressed by Eq (2) whereby the values of λ and v were set at 1500 m and 1 x 10^8^ m/s, respectively [3, 21].

**Fig 3:**
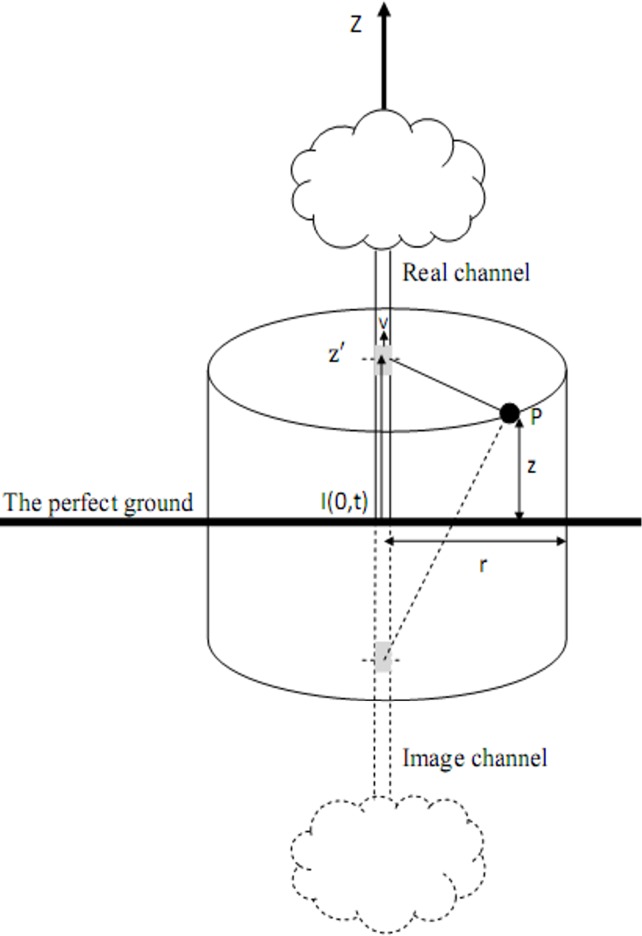
The geometry of the lightning channel.

In addition, the values of the lightning electromagnetic fields and the lightning induced voltage were calculated based on Fig 3, using the analytical expressions as shown in the Eqs (2–4) [1, 3].

$${\text{B}}_{\varphi }\left(\text{r},\text{z},{\text{t}}_{\text{n}}\right)=\sum_{\text{i}=1}^{\text{n}}\sum_{\text{m}=1}^{\text{k}+1}\{{\text{a}}_{\text{m}}{\text{F}}_{\text{i},1}\left(\text{r},\text{z},{\text{t}}_{\text{n}},{\text{h}}_{\text{m},\text{i}}\right)-{\text{a}\prime }_{\text{m}}{\text{F}}_{\text{i},1}\left(\text{r},\text{z},{\text{t}}_{\text{n}},{\text{h}\prime }_{\text{m},\text{i}}\right)\}$$ (2)

$${\text{E}}_{\text{r}}\left(\text{r},\text{z},{\text{t}}_{\text{n}}\right)={\text{E}}_{\text{r}}\left(\text{r},\text{z},{\text{t}}_{\text{n}-1}\right)+\Delta \text{t}\times \sum_{\text{i}=1}^{\text{n}}\sum_{\text{m}=1}^{\text{k}+1}\{{\text{a}}_{\text{m}}{\text{F}}_{\text{i},2}\left(\text{r},\text{z},{\text{t}}_{\text{n}},{\text{h}}_{\text{m},\text{i}}\right)-{\text{a}\prime }_{\text{m}}{\text{F}}_{\text{i},2}\left(\text{r},\text{z},{\text{t}}_{\text{n}},{\text{h}\prime }_{\text{m},\text{i}}\right)\}$$ (3)

$${\text{E}}_{\text{z}}\left(\text{r},\text{z},{\text{t}}_{\text{n}}\right)={\text{E}}_{\text{z}}\left(\text{r},\text{z},{\text{t}}_{\text{n}-1}\right)+\Delta \text{t}\times \sum_{\text{i}=1}^{\text{n}}\sum_{\text{m}=1}^{\text{k}+1}\{{\text{a}}_{\text{m}}{\text{F}}_{\text{i},3}\left(\text{r},\text{z},{\text{t}}_{\text{n}},{\text{h}}_{\text{m},\text{i}}\right)-{\text{a}\prime }_{\text{m}}{\text{F}}_{\text{i},3}\left(\text{r},\text{z},{\text{t}}_{\text{n}},{\text{h}\prime }_{\text{m},\text{i}}\right)\}$$ (4)

Where:

r = radial distance with respect to the lightning channel,

z = height of observation point with respect to the ground surface,

$\vec{B_{\varphi }}$ = magnetic flux density,

$\vec{E_{r}}$ = horizontal electric field,

$\vec{E_{z}}$ = vertical electric field,

$$t_{n}=\frac{\sqrt{r^{2}+z^{2}}}{c}+(n-1)\Delta t\;n=1,2,\ldots ,n_{max}$$

$${\Delta h}_{i}=\left\{ \begin{array}{l} \beta \chi^{2}\{(ct_{i}-ct_{i-1})-\sqrt{{\left(\beta ct_{i}-z\right)}^{2}+{\left(\frac{r}{\chi }\right)}^{2}}+\sqrt{{\left(\beta ct_{i-1}-z\right)}^{2}+{\left(\frac{r}{\chi }\right)}^{2}}\}\;\mathrm{for}\;i>1\\ \beta \chi^{2}\{-(\beta z-ct_{i})-\sqrt{{\left(\beta ct_{i}-z\right)}^{2}+{\left(\frac{r}{\chi }\right)}^{2}}\}\;\mathrm{for}\;i=1 \end{array}\right.$$

$${\Delta h\prime }_{i}=\left\{ \begin{array}{l} \beta \chi^{2}\{(ct_{i-1}-ct_{i})+\sqrt{{\left(\beta ct_{i}+z\right)}^{2}+{\left(\frac{r}{\chi }\right)}^{2}}-\sqrt{{\left(\beta ct_{i-1}+z\right)}^{2}+{\left(\frac{r}{\chi }\right)}^{2}}\}\;\mathrm{for}\;i>1\\ \beta \chi^{2}\{-(\beta z+ct_{i})+\sqrt{{\left(\beta ct_{i}+z\right)}^{2}+{\left(\frac{r}{\chi }\right)}^{2}}\}\;\mathrm{for}\;i=1 \end{array}\right.$$

$$h_{m,i}=\left\{ \begin{array}{l} \frac{({m-1)\times \Delta h}_{i}}{k}+h_{m=k+1,i-1}\\ \frac{({m-1)\times \Delta h}_{i}}{k}\;\mathrm{for}\;i=1 \end{array}\right.$$

$${h\prime }_{m,i}=\left\{ \begin{array}{l} \frac{({m-1)\times \Delta h^{\prime }}_{i}}{k}+{h\prime }_{m=k+1,i-1}\\ \frac{({m-1)\times \Delta h^{\prime }}_{i}}{k}\;\mathrm{for}\;i=1\; \end{array}\right.$$

$$F_{i,1}(r,z,t_{n},h_{m,i})=(\frac{\mu_{0}}{4\pi })\left\{\frac{r}{{\left(\sqrt{r^{2}+{\left(z-h_{m,i}\right)}^{2}}\right)}^{3}}i(h_{m,i},t_{n}-\frac{\sqrt{r^{2}+{\left(z-h_{m,i}\right)}^{2}}}{c})+\frac{r}{{c(\sqrt{r^{2}+{\left(z-h_{m,i}\right)}^{2}})}^{2}}\frac{\partial i(h_{m,i},t_{n}-\frac{\sqrt{r^{2}+{\left(z-h_{m,i}\right)}^{2}}}{c})}{\partial t}\right\}$$

$$F_{i,2}(r,z,t_{n},h_{m,i})=\left(\frac{1}{4\pi \varepsilon_{0}}\right)\left\{\frac{3r(z-h_{m,i})}{{\left(\sqrt{r^{2}+{\left(z-h_{m,i}\right)}^{2}}\right)}^{5}}\times i(h_{m,i},t_{n}-\frac{\sqrt{r^{2}+{\left(z-h_{m,i}\right)}^{2}}}{c})+\frac{3r(z-h_{m,i})}{{c(\sqrt{r^{2}+{\left(z-h_{m,i}\right)}^{2}})}^{4}}\times \frac{\partial i(h_{m,i},t_{n}-\frac{\sqrt{r^{2}+{\left(z-h_{m,i}\right)}^{2}}}{c})}{\partial t}+\frac{r(z-h_{m,i})}{{c^{2}(\sqrt{r^{2}+{\left(z-h_{m,i}\right)}^{2}})}^{3}}\times \frac{\partial^{2}i(h_{m,i},t_{n}-\frac{\sqrt{r^{2}+{\left(z-h_{m,i}\right)}^{2}}}{c})}{\partial t^{2}}\right\}$$

$$F_{i,3}\left(r,z,t_{n},h_{m,i}\right)=\left(\frac{1}{4\pi \varepsilon_{0}}\right)\left\{\frac{2{\left(z-h_{m,i}\right)}^{2}-r^{2}}{{\left(\sqrt{r^{2}+{\left(z-h_{m,i}\right)}^{2}}\right)}^{5}}\times i\left(h_{m,i},t_{n}-\frac{\sqrt{r^{2}+{\left(z-h_{m,i}\right)}^{2}}}{c}\right)+\frac{2{\left(z-h_{m,i}\right)}^{2}-r^{2}}{c{\left(\sqrt{r^{2}+{\left(z-h_{m,i}\right)}^{2}}\right)}^{4}}\times \frac{\partial i\left(h_{m,i},t_{n}-\frac{\sqrt{r^{2}+{\left(z-h_{m,i}\right)}^{2}}}{c}\right)}{\partial t}-\frac{r^{2}}{c^{2}{\left(\sqrt{r^{2}+{\left(z-h_{m,i}\right)}^{2}}\right)}^{3}}\times \frac{\partial^{2}i\left(h_{m,i},t_{n}-\frac{\sqrt{r^{2}+{\left(z-h_{m,i}\right)}^{2}}}{c}\right)}{\partial t^{2}}\right\}$$

$$a_{m}=\left\{ \begin{array}{l} \frac{{\Delta h}_{i}}{2\times k}\;for\;m=1\;and\;m=k+1\\ \;\frac{{\Delta h}_{i}}{k}\;for\;others\; \end{array}\right.$$

$${a\prime }_{m}=\left\{ \begin{array}{l} \frac{{\Delta h^{\prime }}_{i}}{2\times k}\;for\;m=1\;and\;m=k+1\\ \;\frac{{\Delta h^{\prime }}_{i}}{k}\;for\;others\; \end{array}\right.$$

k is division factor (≥2)

**Fig 4:**
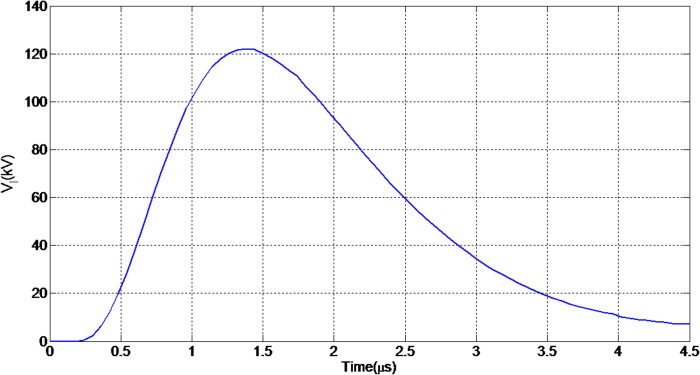
Evaluated induced voltage at r = 50m and h = 10m. The corresponding lightning induced voltage is evaluated assuming there is a lightning strike at 50m away from a 10m height power line.

Fig 4 illustrates the evaluated lightning induced voltage on the line where striking distance is 50m and the conductor height is 10m, respectively. Considering the problem geometry in Figs 1 and 3, and the fact that this particular distance of 50m is within the critical area to cause the flashover, therefore these parameters are used as the basis to carry out the experimental work. Whilst the 1.2/50μs standard lightning impulse voltage waveform generated in the laboratory is to replicate the similarity of the real waveform obtained in natural environment. In addition, the 10m height is to reflect the actual and typical height of an overhead distribution line. Thus, with these several considerations, the peak induced voltage of 122kV obtained is very much reasonable to be chosen and to be injected as a source voltage in experimental work. It is worth to note that the typical Basic Insulation Lightning Level (BIL) for 33 kV distribution line is around 110kV and much less for lower voltage system, such as 22kV or 11kV. That’s mean the higher the voltage above the BIL, the higher the probability of the equipment damages due to this induced voltage.

### Polymer insulator

Insulators, as their name indicates, are used to isolate live conductors from the grounded supporting structure. Insulators are designed with appropriate shapes according to their application. In early times, native materials were used for manufacturing insulators such as porcelain from clay, feldspar and quartz, and glass from silica, soda ash, dolomite, limestone, feldspar and sodium sulphate [22].

Due to material advancement about four decades ago, electrical industries found a solution to overcome the low performance of traditional insulators by introducing the polymer insulator [23]. Indeed, polymer insulators have shown excellent performance under heavily polluted conditions as compared to ceramic and glass types. The superior material also allowed for easy handling and maintenance, e.g. installation and washing. However, the organic compounds of the polymer are much more likely to age in time, mainly caused by electrical overstress and radiation [22–27].

**Fig 5:**
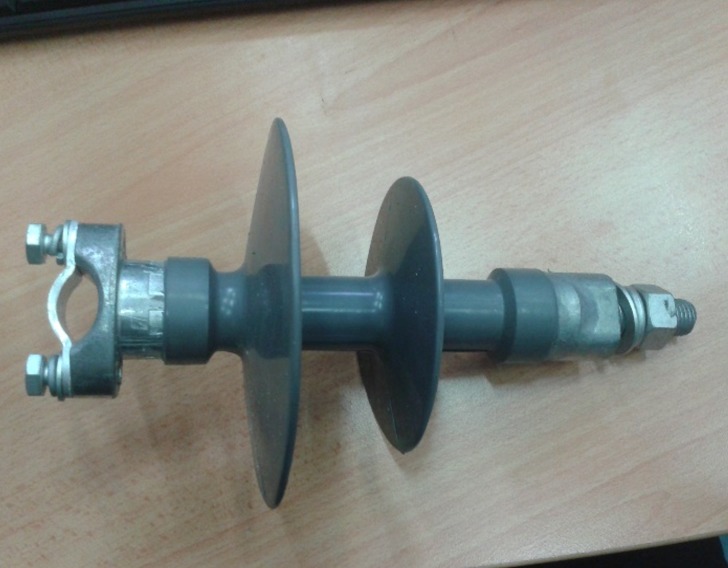
Typical 10kV polymer insulator.

In this study, a 10kV polymer insulator was studied as shown in Fig 5 and the specifications of the insulator are tabulated in Table 2 as follows:

**Table 2:** The specifications of a 10kV polymer insulator.

Parameter	Value
**Rated Voltage (kV)**	10
**Rated Mechanical Load (kN)**	4
**Structure Height (mm)**	250
**Min Arcing distance (mm)**	165
**Min Nominal Creepage Distance (mm)**	420
**Shed Diameter (mm)**	148/118

**Fig 6:**
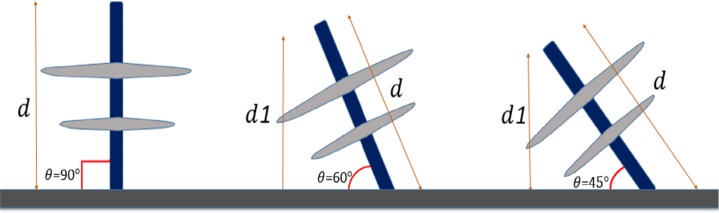
Insulator inclination during the service life.

It is known that the insulator was prone to environmental degradation. Perhaps, the mechanical characteristic of the insulator is highly at risk. Occasionally, some insulator will bend due to the weight load of the power line or results of the external force like a fallen tree or faulty pole. On field observation has recorded that the insulators were inclined (as shown in Fig 6) due to the events which consequently influence the clearance of the line to the nearby grounded parts such as cross-arm.

**Fig 7:**
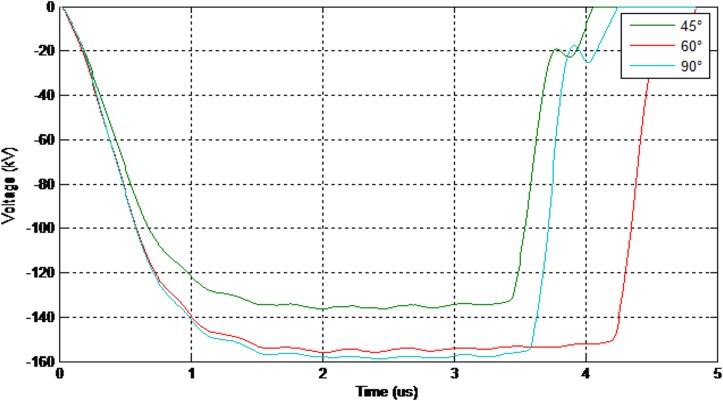
The insulator angle with respect to the cross-arm. Insulator inclined at such angle with respect to the cross-arm due to physical stresses during the service life.

In this research, the values for electrical breakdown under a negative and positive standard impulse voltage (1.2/50 μs) was investigated assuming weather conditions of wet and wet with 4% salt. In order to represent the effect of the insulator angle with respect to the cross-arm (due to the force axis on the line as shown in Fig 7), a metal grounded plate was set at the bottom of the insulator and the electrical behaviour of the insulator under different angles with respect to the metal plate was investigated and the values of the leakage current and breakdown voltage were determined.

**Fig 8:**
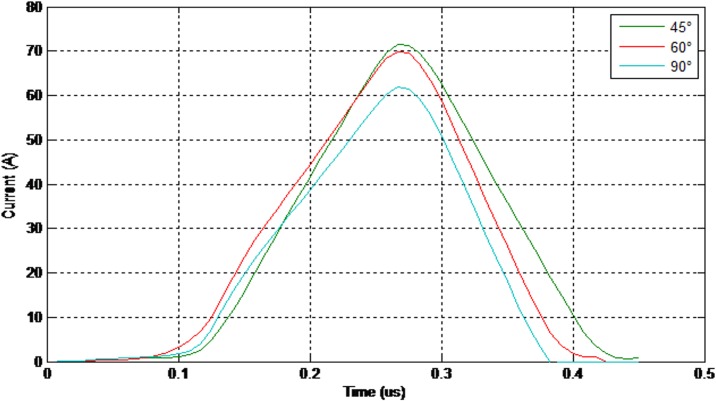
The values of the breakdown voltage (negative impulse-wet condition).

**Fig 9:**
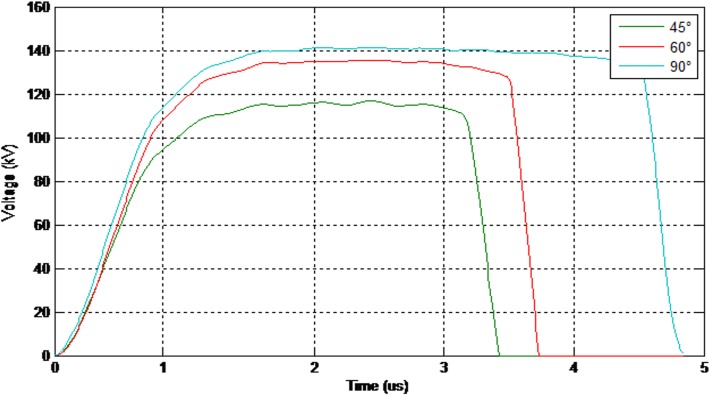
The values of the leakage current (negative impulse-wet condition).

Figs 8 and 9 show the values of the 50% breakdown voltage and leakage current for different angles with respect to the cross arm under a negative impulse voltage (1.2/50μs) and wet conditions.

**Fig 10:**
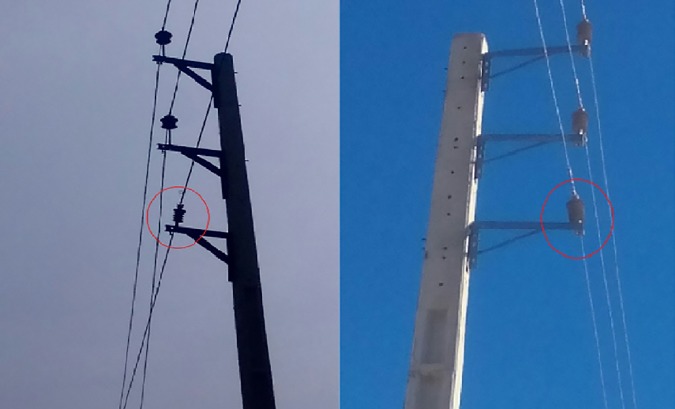
The values of the breakdown voltage (positive impulse-wet condition).

Moreover, Fig 10 illustrates the value of the 50% breakdown voltage for different angles with respect to the cross arm under a positive impulse voltage (1.2/50μs) and wet conditions.

Referring to Fig 10, by applying such smaller angles to the insulator, the breakdown voltage decreased significantly. It has been noticed that the time taken for breakdown also shorten within 1.5 μs which is noteworthy for lightning protection study.

**Fig 11:**
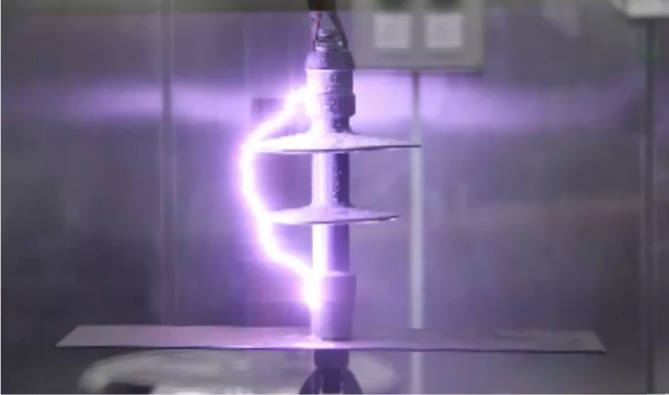
The electrical breakdown for 90° angle with respect to the cross-arm.

**Fig 12:**
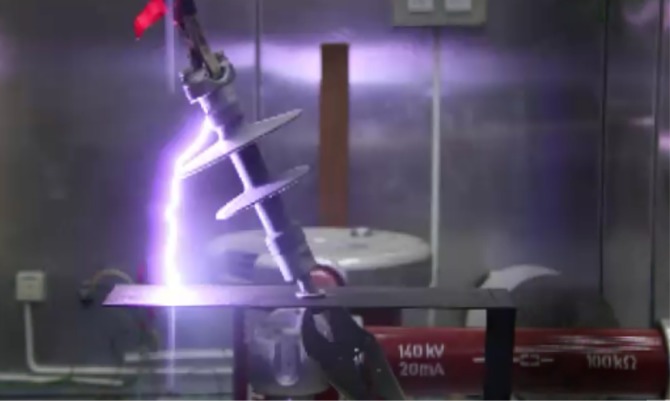
The electrical breakdown for 60° degrees angle with respect to the cross-arm.

Figs 11 and 12 show the electrical breakdown for different insulator angles with respect to the cross-arm. Both figures indicate the different path of flashover due to the changes of clearance distance.

**Fig 13:**
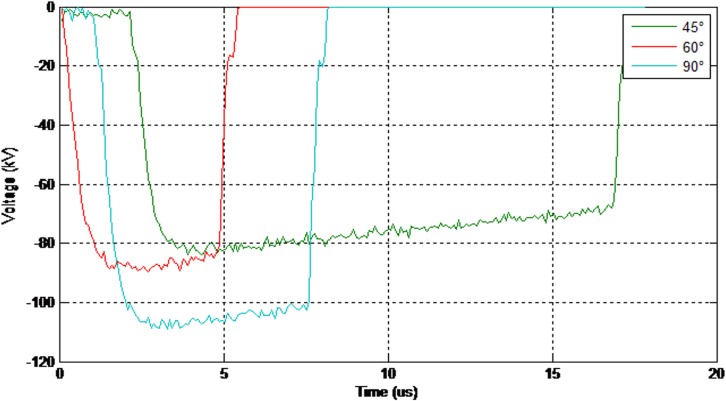
The values of the breakdown voltage (negative impulse-wet with 4% salt condition).

**Fig 14:**
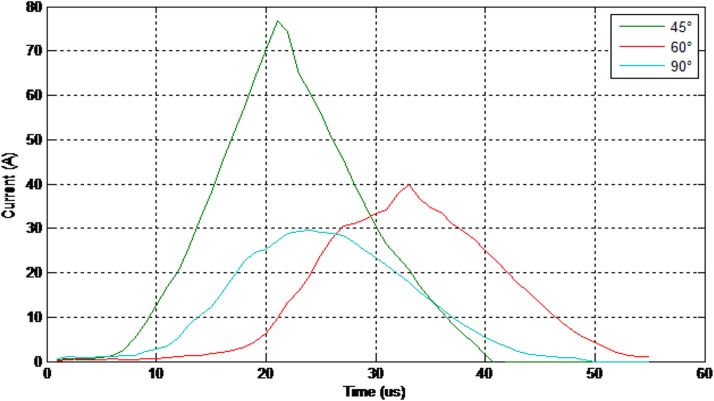
The values of the leakage current (negative impulse-wet with 4% salt condition).

As illustrated in Figs 11 and 12 the electrical discharge path for 90° is different from 60° because of an increase in the electric field in the area between the insulator and the cross-arm. The values of the 50% breakdown voltage and leakage current for different angles with respect to the cross arm under a negative impulse voltage (1.2/50 μs) and wet conditions with 4% salt conditions is demonstrated in Figs 13 and 14.

**Fig 15:**
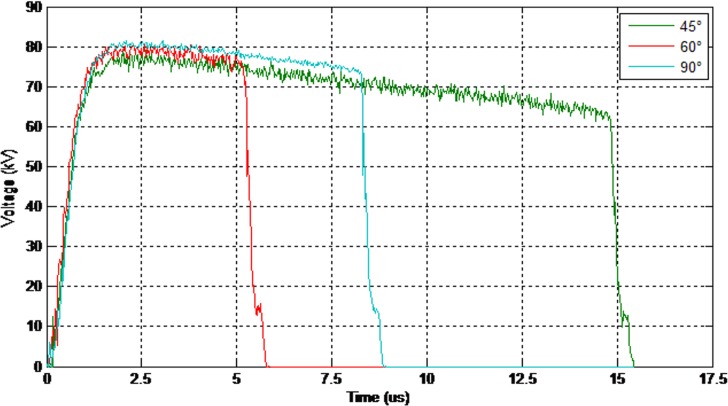
The values of the breakdown voltage (positive impulse-wet with 4% salt condition).

**Fig 16:**
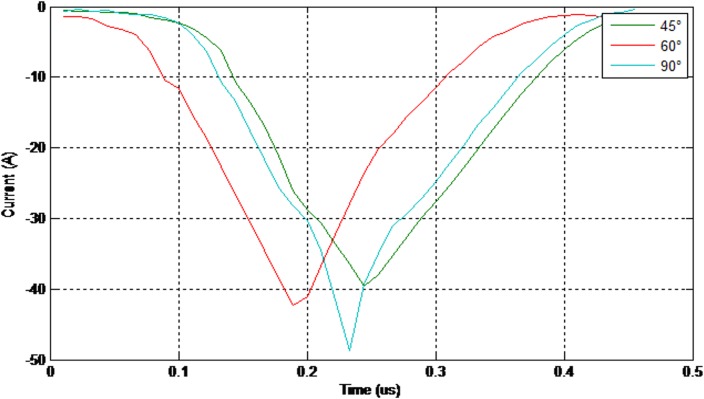
The values of the leakage current (positive impulse-wet with 4% salt condition).

Moreover, the values of the 50% breakdown voltage and leakage current for different angles with respect to the cross arm under negative impulse voltage (1.2/50μs) and wet with 4% salt conditions are shown in Figs 15 and 16.

**Fig 17:**
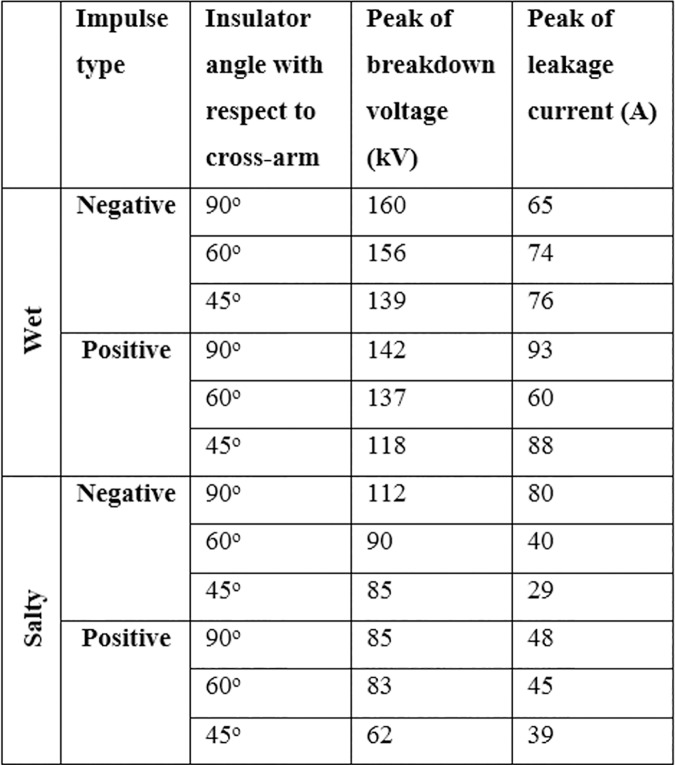
The peak values of the breakdown voltage and leakage current. The presented values of breakdown voltage are the average values of 10 impulse withstand tests and the obtained data are available in S1 Appendix.

Fig 17 shows the peak values of the breakdown voltage and leakage current under different impulses, insulator angle and wet conditions. The results show that the insulator angle with respect to the cross-arm has an effect on the values of electrical breakdown for both positive and negative impulses and also under both wet and salty wet conditions. This is because of changes in the field distribution in the space between the insulator and the cross-arm. Moreover, by reducing the insulator angle, the values of the leakage current at breakdown show a reducing trend.

### Electrical modelling of a polymer insulator

**Fig 18:**
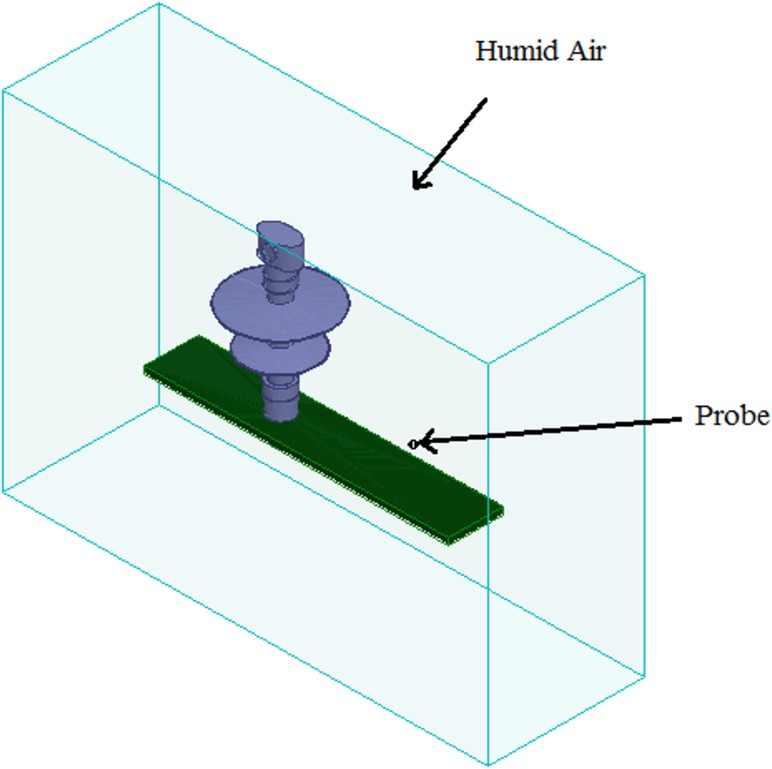
Simulation of insulator based on experimental work. A real insulator was simulated in FEM based software accordingly to experimental work. The insulator was changed to several angles and the electrical profile of the insulator was observed at the probe location.

**Fig 19:**
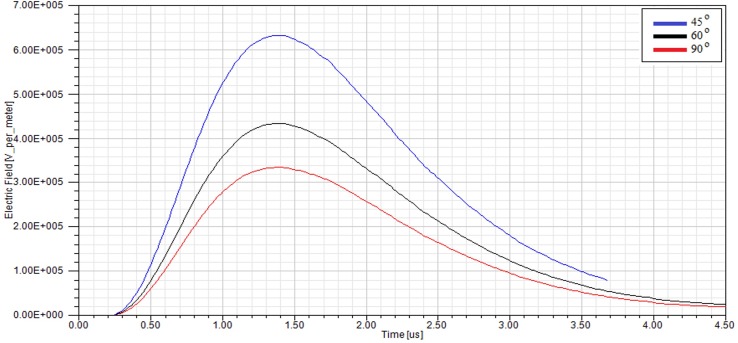
The profile of the electric field under a lightning impulse voltage at different insulator angles for dry conditions.

**Fig 20:**
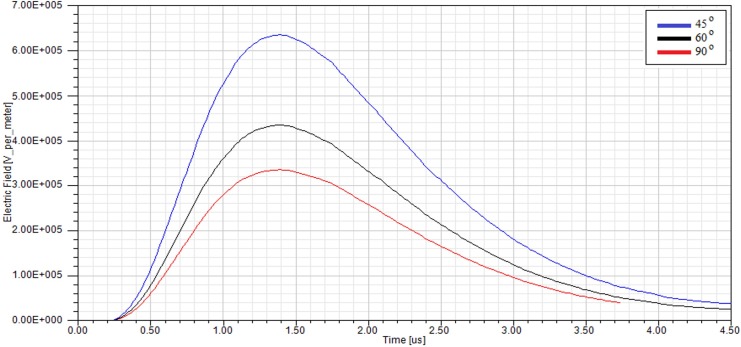
The profile of the electric field under a lightning impulse voltage at different insulator angles for humid conditions.

In order to consider the electrical behaviour of an insulator under different inclination angles, the finite element analysis (FEA) method was used. This method assisted the efficient analysis of field distributions across different materials. A full scale model of a 10 kV polymer insulator was modelled using the Ansys HFSS software. Fig 18 shows the model attached to a cross-arm structure and surrounded by air. In addition, Fig 19 shows the profile of the electric field versus time for three different insulator angles (angles with respect to the cross-arm) during dry conditions while Fig 20 shows the same simulation but under humid conditions. The model was energised by a lightning induced voltage as presented in Fig 4 which recorded as high as 122 kV. The measurement of the electric fields was taken at the reference point marked as “probe”.

**Fig 21:**
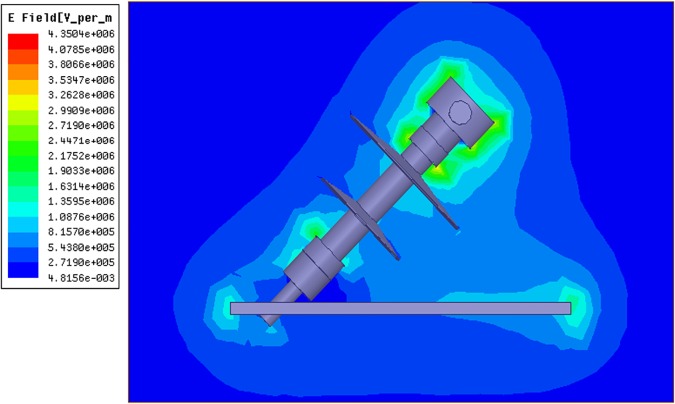
Electric fields distribution on inclined insulator.

As shown in Figs 19 and 20, the insulator angle has a significant effect on the profile of the electric field within the space near to the insulator, especially for the smaller angles. Note that the electric field increased for the smaller angles because of the shorter live-to-ground distance which means the possibility of flashover occurring is increased. Similarly, the rate of degradation of the polymer insulator could be amplified. Fig 21 shows the electric field distribution within the space around the insulator with an inclination angle. The maximum and mean values of the electric field in the space between the insulator and cross-arm for different angles are summarised accordingly in Table 3.

**Table 3:** The values of the electric field for different insulator angles with respect to the cross-arm.

Angle with respect to cross-arm	Dry		Humid	
	E_max_ (kV/m)	E_mean_ (kV/m)	E_max_ (kV/m)	E_mean_ (kV/m)
90°	335.39	134.67	337.81	164.76
60°	434.00	174.24	436.66	174.41
45°	632.23	313.97	635.12	254.63

The results show that the insulator angle can effectively escalate the field distribution in the space between the insulator and the cross-arm regardless of the air humidity. It was calculated that the percentage difference of the electric field (between 90° and 45°) can be up to 88%. The significant increase of the electric field due to the small inclination angle reduces the electrical breakdown values as the ionisation of the air takes place at a lower voltage. Moreover, Table 3 also indicates that the value of the electric field at the reference point is slightly increased by about 0.4% - 0.7% of difference due to changes in humidity in the surrounding air. Particularly, the electric field increases when the humid air has a high volume conductivity compared to dry air.

## Discussion

**Fig 22:**
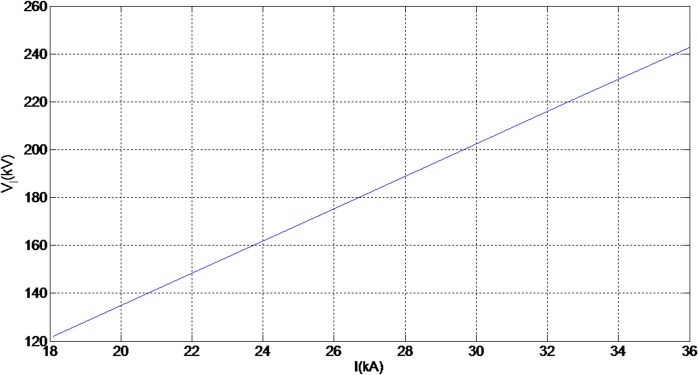
Behaviour of lightning induced voltage versus current peak changes (r = 50m, h = 10m).

**Fig 23:**
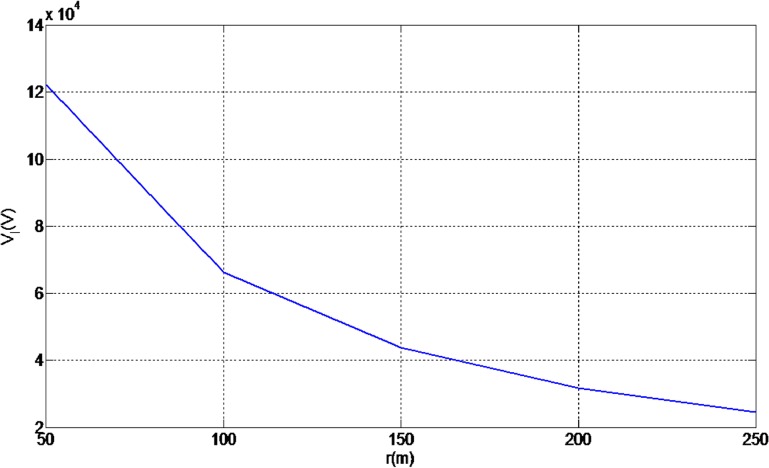
Behaviour of lightning induced voltage versus radial distance changes (Ip = 18.1kA, h = 10m).

Lightning induced voltage is one of the major electrical problems for MV lines because of the length of the line and the low value of the CFO of the line compared to transmission lines. On the other hand, when considering the indirect effects of lighting, the lightning electromagnetic field is the source of the lightning induced voltage which can be created by any lightning events around the line [11, 28, 29]. Therefore, in distribution networks the chance of indirect effects is higher than direct effects. Moreover, in order to design appropriate values for lightning protection and also to increase the insulation level of the line, consideration of the weather conditions, the structure of line and also the insulator angle will be required, otherwise neglecting these factors will cause the stability of the line to be reduced. It is important to take account of the peak value of the lightning induced voltage in Fig 4 and also the behaviour of the lightning induced voltage versus the current and radial distance changes (as illustrated in Figs 22 and 23 respectively). Based on Fig 22 the induced voltage on the power line increases linearly with the peak current, thus indicating that serious measures should be taken when designing both the current and voltage withstand capabilities of electrical systems.

The behaviour of the lightning induced voltage presented in Fig 22 is dependently changes according to the striking distance. In contrast, the results in Fig 23 depicts a decreasing trend of induced voltage over the striking distance, r.

This work studied the behaviour of a polymer insulator under different weather conditions and also insulator angles (with respect to the cross-arm) and can be helpful to set proper lightning protection on a MV line. Ultimately, the high lightning density in Malaysia requires more research in this field in order to optimise the entire system to avoid costly failure in advance.

## Conclusion

In this paper, the electrical performance of a 10 kV polymer insulator was determined experimentally under negative and positive impulse conditions for different types of weather and insulator angles. Moreover, the behaviour of the electric field and voltage along an insulator for different insulator angles was studied by modelling the insulator and the results were discussed appropriately. Results showed that the insulator angle and weather conditions reduced the value of the breakdown voltage and thus will reduce the line’s lightning performance. Furthermore, this behaviour provides excellent information on what should be done to protect those lines against the indirect effects of lightning, whereby consideration should be given to different impulse voltages and also the use of local information of the environment.

## Supporting information

S1 Appendix (DOCX)
