# Supplementary material for: The influence of lightning induced voltage on the distribution power line polymer insulators
Source: PLoS One. 2017 Feb 24;12(2):e0172118. doi: 10.1371/journal.pone.0172118 (PMC5325478; doi:10.1371/journal.pone.0172118)
Supplement: S1 Appendix — (DOCX) [file pone.0172118.s001.docx]

**S1 Appendix: Experimental Results**

In the study, voltage rising method was applied for impulse withstand test. Ten trials were acquired to complete an individual case study where the average value of breakdown is calculated. Table 1 and 2 listed the data collected in both experiments in wet and salty conditions and different polarity conditions.

**Table 1: Breakdown Voltages; Condition: Wet**

| **Trials** | **Breakdown Voltage (kV)** | | | | | | | | | | |
| --- | --- | --- | --- | --- | --- | --- | --- | --- | --- | --- | --- |
|  | **90º** | | | **60º** | | | | **45º** | | | |
|  | **+VE** | | **-VE** | **+VE** | | **-VE** | | **+VE** | | | **-VE** |
| 1 | 144.03 | | 161.07 | 135.02 | | 153.18 | | 121.75 | | | 142.43 |
| 2 | 148.83 | | 151.31 | 133.43 | | 152.31 | | 120.67 | | | 139.28 |
| 3 | 139.23 | | 161.07 | 132.23 | | 158.34 | | 115.02 | | | 136.53 |
| 4 | 129.63 | | 161.07 | 136.14 | | 156.21 | | 118.05 | | | 141.06 |
| 5 | 139.23 | | 151.31 | 140.24 | | 157.03 | | 123.18 | | | 142.27 |
| 6 | 144.03 | | 165.95 | 136.06 | | 162.02 | | 116.26 | | | 139.01 |
| 7 | 134.43 | | 161.07 | 138.42 | | 154.13 | | 113.23 | | | 139.79 |
| 8 | 139.23 | | 161.07 | 145.02 | | 162.59 | | 118.25 | | | 135.51 |
| 9 | 148.83 | | 156.19 | 132.13 | | 157.98 | | 119.98 | | | 132.36 |
| 10 | 148.83 | | 170.84 | 138.01 | | 150.61 | | 116.01 | | | 136.98 |
|  |  |  | | |  | |  | |  |  | |
| **Av. (kV)** | 141.63 | | 160.10 | 136.67 | | 156.44 | | 118.24 | | | 138.52 |
| **Std. Dev** | 6.51 | | 6.00 | 3.97 | | 3.98 | | 3.16 | | | 3.19 |
| **Rounded Value (kV)** | 142 | | 160 | 137 | | 156 | | 118 | | | 139 |

*****Atmospheric correction was applied.

**Table 2: Breakdown Voltages; Condition: Salty**

| **Trials** | **Breakdown Voltage (kV)** | | | | | | | | | | |
| --- | --- | --- | --- | --- | --- | --- | --- | --- | --- | --- | --- |
|  | **90º** | | | **60º** | | | | **45º** | | | |
|  | **+VE** | | **-VE** | **+VE** | | **-VE** | | **+VE** | | | **-VE** |
| 1 | 78.10 | | 107.73 | 86.21 | | 97.35 | | 65.22 | | | 82.35 |
| 2 | 82.98 | | 117.53 | 82.04 | | 92.18 | | 60.40 | | | 82.71 |
| 3 | 92.74 | | 122.43 | 88.90 | | 93.29 | | 57.71 | | | 86.44 |
| 4 | 78.10 | | 102.84 | 79.23 | | 84.25 | | 65.39 | | | 87.00 |
| 5 | 87.86 | | 107.73 | 84.59 | | 87.39 | | 53.52 | | | 88.22 |
| 6 | 82.98 | | 112.63 | 82.38 | | 88.42 | | 61.64 | | | 84.91 |
| 7 | 82.98 | | 107.73 | 78.02 | | 85.37 | | 60.90 | | | 87.73 |
| 8 | 89.86 | | 117.53 | 74.67 | | 89.80 | | 62.35 | | | 85.33 |
| 9 | 82.98 | | 112.63 | 85.57 | | 90.40 | | 67.56 | | | 81.96 |
| 10 | 87.86 | | 107.73 | 84.23 | | 94.35 | | 63.02 | | | 83.42 |
|  |  |  | | |  | |  | |  |  | |
| **Av. (kV)** | 84.64 | | 111.65 | 82.584 | | 90.28 | | 61.77 | | | 85.01 |
| **Std. Dev** | 4.83 | | 6.02 | 4.27 | | 4.12 | | 4.05 | | | 2.31 |
| **Rounded Value (kV)** | 85 | | 112 | 83 | | 90 | | 62 | | | 85 |

*****Atmospheric correction was applied.
